# Editorial: New insights into the relationship between endocrine disorders and dental diseases

**DOI:** 10.3389/fendo.2025.1560318

**Published:** 2025-02-11

**Authors:** Chaofan Zhang, Shan Jiang, Nianye Zheng, Yunzhi Lin

**Affiliations:** ^1^ Department of Orthopaedic Surgery, the First Affiliated Hospital, Fujian Medical University, Fuzhou, China; ^2^ Department of Orthopaedic Surgery, National Regional Medical Center, Binhai Campus of the First Affiliated Hospital, Fujian Medical University, Fuzhou, China; ^3^ Fujian Provincial Institute of Orthopedics, the First Affiliated Hospital, Fujian Medical University, Fuzhou, China; ^4^ Fujian Orthopedic Bone and Joint Disease and Sports Rehabilitation Clinical Medical Research Center, Fuzhou, China; ^5^ Shenzhen Stomatology Hospital (Pingshan), Southern Medical University, Shenzhen, China; ^6^ Department of Orthopedic Surgery, Washington University in St. Louis, St. Louis, MO, United States; ^7^ Department of Stomatology, the First Affiliated Hospital, Fujian Medical University, Fuzhou, China; ^8^ Department of Stomatology, National Regional Medical Center, Binhai Campus of the First Affiliated Hospital, Fujian Medical University, Fuzhou, China

**Keywords:** endocrine disorders, dental diseases, bone remodelling, adipokine, endocrine factors

Endocrine disorders such as diabetes, metabolic syndrome, and thyroid dysfunctions have been associated with an increased risk for various dental diseases. However, many aspects of these relationships remain to be elucidated. This Research Topic aimed to provide new insights into the connections between endocrine disorders and dental pathologies.

Yang et al.’s study conducted a comprehensive cross-sectional study involving 2,530 euthyroid adults, revealing that reduced sensitivity to thyroid hormones (THs) is associated with a heightened risk of periodontitis. Their findings identify serum 25-hydroxyvitamin D (25[OH]D) as a mediator in this relationship, suggesting that maintaining adequate levels of 25(OH)D may help modulate thyroid hormone sensitivity and subsequently reduce the risk of periodontitis. This mediation likely operates through 25(OH)D’s role in modulating inflammatory pathways and supporting thyroid hormone homeostasis. These insights call for further research to establish causality and explore the therapeutic potential of vitamin D optimization in improving thyroid hormone function and periodontal health.

Furthermore, the study by Yan et al. illuminates the complex interactions between progesterone supplementation and periodontal health in perimenopausal women. Specifically, the research focused on oral administration of 10 mg dihydroxyprogesterone tablets taken twice daily for 10 days per month over a 6-month period. This targeted progesterone intervention demonstrated a context-dependent inflammatory response: enhancing periodontal inflammation in the absence of scaling and root planing (SRP), yet dampening inflammatory markers when combined with SRP. The findings underscore the nuanced role of hormone supplementation in managing periodontal conditions during the perimenopausal transition.”

Additionally, debris from extracted teeth without active pathology showed promise as bone graft expanders supporting regeneration, while promoting gingival healing and tissue integration (Xu et al.). The study specifically used premolars extracted for orthodontic reasons from patients with healthy periodontal tissues and no systemic diseases. Further optimization warrants exploring whether inherent signals may be leveraged to improve outcomes.

Finally, poor oral health marked by periodontitis and tooth loss associated with increased osteoporotic fractures, whereas routine dental care and hygiene linked to reduced fracture hazards (Park et al.). This reinforces the necessity of coordinated management across dental and skeletal health to mitigate systemic bone complications.

Collectively, these findings significantly expanded the current understanding on the relationship between endocrine disorders and dental diseases. Ongoing efforts to translate these findings into coordinated screening and early preventive interventions across medicine and dentistry are critical to alleviate long-term oral-systemic morbidities. Continued mechanistic exploration of connections between endocrine derangements and compromised dental health remains imperative.

